# Personalized Response to Empagliflozin in Heart Failure: Association of BDNF and ATP2A2 Variants in a South Asian Cohort

**DOI:** 10.3390/biomedicines13092095

**Published:** 2025-08-28

**Authors:** Qura Tul Ain, Abida Shaheen, Umer Ijaz, Sagheer Ahmed, Muhammad Usman, Mushood Ahmed, Muhammad Ali, Fahad Azam, Asaad Akbar Khan, Ali Hasan, Raheel Ahmed

**Affiliations:** 1 Department of Pharmacology, Shifa College of Medicine, Shifa Tameer-e-Millat University, Islamabad 44000, Pakistan; qura-tul-ain.scm@stmu.edu.pk (Q.T.A.); abida.scm@stmu.edu.pk (A.S.); fahad.scm@stmu.edu.pk (F.A.); 2 Department of Cardiology Shaukat Khanum Memorial Cancer Hospital and Research Centre, Lahore 54782, Pakistan; uijaz07@gmail.com; 3 Department of Basic Health Sciences, Shifa College of Pharmaceutical Sciences, Shifa Tameer-e-Millat University, Islamabad 44000, Pakistan; sagheer.scps@stmu.edu.pk; 4 Department of Gastroenterology Services Hospital, Lahore 54000, Pakistan; mu97950@gmail.com; 5 Department of Medicine, Rawalpindi Medical University, Rawalpindi 46000, Pakistan; mushood07@gmail.com; 6 Department of Emergency Medicine, Shifa International Hospital, Islamabad 44000, Pakistan; kenfinder89@gmail.com; 7 Department of Cardiology, Shifa International Hospital, Islamabad 44000, Pakistan; asaad.akbar@gmail.com; 8 Department of Cardiology, Imperial College London, London SW7 2AZ, UK; r.ahmed21@imperial.ac.uk; 9 Department of Cardiology, Royal Brompton Hospital, London SW3 6NP, UK

**Keywords:** empagliflozin, heart failure, pharmacogenetics, *BDNF* rs6265, *ATP2A2* rs1860561, ejection fraction, BNP, SGLT2 inhibitors

## Abstract

**Background:** Empagliflozin, a sodium–glucose cotransporter 2 (SGLT2) inhibitor, improves outcomes in heart failure (HF) patients, yet inter-individual variability in response remains unclear. Genetic variants in Brain-Derived Neurotrophic Factor *BDNF* (rs6265) and ATPase Sarcoplasmic/Endoplasmic Reticulum Ca^2+^ Transporting 2 ATP2A2 (rs1860561) may influence the treatment efficacy. **Objective:** To assess the association of *BDNF* and *ATP2A2* polymorphisms with the response to low-dose empagliflozin (10 mg) in Pakistani patients with heart failure and a reduced ejection fraction (HFrEF). **Methods:** In this prospective study, 120 HF patients with an ejection fraction of 25–45% who had been on stable standard heart failure therapy for at least 3 months were initiated on 10 mg of empagliflozin. The brain natriuretic peptide (BNP) and LVEF left ventricular ejection fraction (LVEF) were assessed at 6 and 12 months. Genotyping for rs6265 and rs1860561 was performed via Sanger sequencing. A response was defined as a ≥5% EF increase or ≥20% BNP reduction. Associations were analyzed using chi-square and logistic regression. **Results:** Among 99 genotyped patients, *BDNF* T allele carriers (CT/TT) had a significantly lower EF (*p* = 0.028) and BNP (*p* < 0.001) response. The CC genotype was associated with improved outcomes (BNP OR: 7.70; EF OR: 5.97). For *ATP2A2*, the GG genotype showed a strong association with EF improvement (OR: 5.97; *p* = 0.001), with no BNP association. Variant allele frequencies were higher among Punjabis and Kashmiris than Pathans. **Conclusions:** BDNF rs6265 and ATP2A2 rs1860561 polymorphisms appear to influence the individual response to empagliflozin in HFrEF patients. These findings underscore the potential of pharmacogenetic profiling to guide personalized therapy and optimize treatment outcomes in heart failure.

## 1. Introduction

Empagliflozin, a sodium–glucose cotransporter 2 inhibitor, has become a cornerstone in the treatment of heart failure in patients with a both reduced ejection fraction and preserved ejection fraction [1,2]. Compared to other HF agents, empagliflozin uniquely improves renal and cardiac outcomes independent of glycemic control, making it an ideal candidate for pharmacogenetic analysis [3]. Despite these broad benefits, the therapeutic response to empagliflozin remains heterogenous. Variability in clinical endpoints such as changes in the left ventricular ejection fraction (LVEF) and brain natriuretic peptide (BNP) levels suggests underlying biological differences, possibly rooted in the patient-specific genetic makeup [4,5].

Recent studies have investigated the effects of low-dose empagliflozin (EMPA) and other treatments on heart failure patients. EMPA, when combined with standard heart failure therapy, significantly improved the ejection fraction and reduced N-terminal pro-brain natriuretic peptide (NT-proBNP) levels compared to metformin in patients with mildly reduced EF [6]. Although empagliflozin led to reductions in heart failure events and hospitalizations in some cases, it did not produce a significant improvement in the ejection fraction [7]. These findings suggest that the benefits of empagliflozin in heart failure patients may not be primarily driven by changes in EF and BNP [5]. These discrepancies underscore the need to explore pharmacogenetic factors that may govern individual variability in the drug response [8]. To date, the pharmacogenetic landscape of SGLT2 inhibitors has predominantly centered on SLC5A2, the gene encoding the SGLT2 protein [9]. While polymorphisms in SLC5A2 offer mechanistic insight into the transporter function, they do not fully account for downstream myocardial signaling, structural remodeling, or calcium handling processes that are critically involved in heart failure pathophysiology and may modulate the treatment response [10,11]. Moreover, earlier studies have largely overlooked diverse ethnic cohorts, particularly South Asian populations who face a disproportionately high burden of heart failure. These discrepancies underscore the need to explore pharmacogenetic factors that may govern individual variability in the drug response [8]. The pharmacodynamic effects of a low dose of 10 mg of empagliflozin can be modulated by various genetic polymorphisms, particularly in genes that regulate the cardiac function and metabolic pathways [12,13]. Two genes of interest in this context are Brain-Derived Neurotrophic Factor (BDNF) and ATPase Sarcoplasmic/Endoplasmic Reticulum Ca2+ Transporting 2 (ATP2A2) [14,15]. One such gene is BDNF (brain-derived neurotrophic factor), where the Val66Met polymorphism (rs6265) has been linked to altered cardiac remodeling and neurotrophic signaling, which influences the heart function [14]. Elevated levels of circulating BDNF are associated with improved cardiomyocyte survival, and variations in BDNF may play a role in how patients respond to treatment [16]. Recent studies suggest that the Val66Met polymorphism may impact the response to heart failure therapies, due to its role in modulating heart health and treatment efficacy [17].

Moreover, genetic variations in the ATP2A2 gene, which encodes the sarcoplasmic/endoplasmic reticulum Ca2+-ATPase (SERCA2a), can influence calcium handling in cardiomyocytes, a critical process for heart muscle contraction and relaxation [18]. The rs1860561 polymorphism in ATP2A2 may alter SERCA2a activity, potentially affecting calcium regulation in heart cells [19]. Given its influence on cardiac function, rs1860561 may play a role in modulating the response to empagliflozin in patients with heart failure.

This study investigates whether the BDNF Val66Met (rs6265) and ATP2A2 rs1860561 polymorphisms are associated with a differential therapeutic response to low-dose empagliflozin in patients with heart failure. By exploring the pharmacogenetic basis of treatment variability, the findings may support more individualized therapeutic strategies in cardiovascular care.

## 2. Materials and Methods

### 2.1. Study Design and Population

A total of 120 patients with heart failure were enrolled in this prospective, multicenter observational cohort study conducted in Pakistan from 2023 to 2024. All patients provided written informed consent prior to enrollment. The study protocol received ethical approval from the Institutional Review Board (IRB# 089-24) and was conducted in accordance with the Declaration of Helsinki and Good Clinical Practice (GCP) guidelines.

### 2.2. Inclusion and Exclusion Criteria

Participants aged 18 to 75 years with a documented diagnosis of heart failure (HF) and a left ventricular ejection fraction (LVEF) between 25% and 45% were considered eligible for inclusion. Inclusion also required an elevated B-type natriuretic peptide (BNP) level of ≥300 pg/mL or ≥900 pg/mL in patients with atrial fibrillation. All participants were required to have been on stable, guideline-directed medical therapy comprising beta-blockers, ACE inhibitors/ARBs/ARNIs, and mineralocorticoid receptor antagonists for at least the preceding 3 months. Additional eligibility criteria included adequate cognitive function, the ability to provide informed consent, and willingness to comply with a study follow-up. Patients were excluded if they had severe renal impairment (eGFR < 30 mL/min/1.73 m^2^), known hypersensitivity or contraindications to empagliflozin or dapagliflozin, were pregnant or lactating, had been recently hospitalized or had medication changes within the last three months, had comorbidities known to significantly affect BNP levels (e.g., severe liver disease), were participating in another clinical trial, or were unable to adhere to the study requirements.

### 2.3. Clinical Parameter Assessment

BNP levels and LVEF were measured at the baseline, 6 months, and 12 months. BNPs were measured using a standardized immunoassay method, performed on plasma samples collected via peripheral venipuncture and processed within 2 h of collection. Echocardiography was performed by certified cardiologists using standard transthoracic two-dimensional techniques, in accordance with the American Society of Echocardiography (ASE) guidelines. LVEF was estimated using the modified Simpson’s biplane method.

### 2.4. Sample Collection, DNA Extraction, and Genotyping

Peripheral blood (5 mL) was collected in EDTA tubes and stored at 4 °C or −80 °C. Genomic DNA was extracted using the phenol–chloroform method. DNA purity and concentration were assessed via Nanodrop spectrophotometry (A260/A280 ratio of 1.8–2.0 deemed acceptable), and integrity was confirmed using 1% agarose gel electrophoresis.

The genotyping of BDNF (rs6265, C > T) and ATP2A2 (rs1860561, G > A) was conducted using PCR followed by Sanger sequencing. The gene-specific primers used were as follows:

- BDNF (rs6265):

Forward: ATTCTCTTAATCCCCGAACTCAACCT

Reverse: GCCAAGATGTGCCTTTGGGAATGTA

- ATP2A2 (rs1860561):

Forward: ATTCACCCCTAGTCTCTCTAGT

Reverse: TGATCCCACTTTCATCCTGGGC

The PCR products were purified (Zymo DNA Clean & Concentrator™-25, Beijing TSINGKE Xinye Biotechnology technology Company, Wuqing District, China) and sequenced using the ABI PRISM BigDye Terminator v3.1 kit. Sequencing was performed on an ABI PRISM 3730XL DNA Analyzer and genotypes were analyzed using FinchTV software v1.4.0.

### 2.5. Endpoints

The primary endpoint of this study was to evaluate the association between genetic polymorphisms in BDNF (rs6265, C > T) and ATP2A2 (rs1860561, G > A) and the clinical response to low-dose empagliflozin (10 mg once daily) in patients with heart failure with a reduced ejection fraction. The clinical response was assessed based on changes in the BNP levels and LVEF, measured at 6 and 12 months after empagliflozin initiation. The reference values for the BNP level and LVEF were obtained after three months of optimized standard heart failure therapy, immediately prior to empagliflozin administration. A reduction of ≥20% in BNPs was defined as a BNP response, while an absolute increase of ≥5% in LVEF from the reference measurement was considered an LVEF response. Secondary endpoints included changes in BNP levels and LVEF at 12 weeks and 6 months, and the comparison of the treatment response across different genotypic groups. The association between these polymorphisms and clinical response was further evaluated using genotype-stratified analyses and logistic regression models, adjusting for potential clinical confounders such as ischemic heart disease, hypertension, gender, and smoking status.

### 2.6. Statistical Analysis

All statistical analyses were conducted using IBM SPSS Statistics version 23. Continuous variables were expressed as mean ± standard deviation and categorical variables as frequencies and percentages. Paired t-tests were used to evaluate changes in BNP levels and LVEF at 6 and 12 months compared to the baseline. Independent t-tests were applied to compare continuous variables across genotype subgroups.

Chi-square tests were used to assess associations between the genotype and response categories, defined as a ≥20% reduction in BNPs and ≥5% absolute increase in LVEF. Pearson’s correlation was used to evaluate the relationship between BNP reduction and LVEF improvement.

To identify independent predictors of the treatment response, multivariate logistic regression models were constructed. The response (BNP or LVEF) was used as the dependent variable, while the genotype (BDNF rs6265 or ATP2A2 rs1860561) was the primary independent variable. Covariates including age, gender, hypertension, ischemic heart disease, and smoking status were included to adjust for potential confounding. Adjusted odds ratios (ORs) with 95% confidence intervals (CIs) were reported. A two-sided *p*-value < 0.05 was considered statistically significant.

## 3. Results

### 3.1. Demographics and Clinical Characteristics

**Figure 1:**
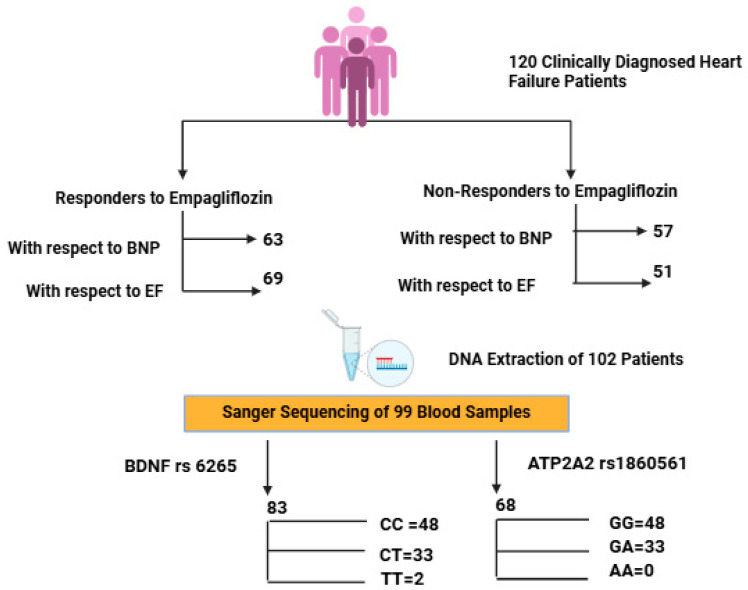
Flowchart depicting patient stratification based on clinical response to empagliflozin and subsequent genotyping analysis of *BDNF* (rs6265) and *ATP2A2* (rs1860561) polymorphisms. Created with BioRender.com, in accordance with BioRender copyright.

A total of 120 patients were enrolled in this study. Of these, 69 patients (57.5%) were categorized as EF responders based on an improvement in their ejection fraction, while 51 patients (42.5%) did not demonstrate improvement. Classification based on BNP levels showed 63 responders (52.5%) and 57 non-responders (47.5%), as shown in Figure 1. The mean age was consistent across all subgroups, ranging from 58 to 59 years. Male participants were predominant in both the EF (68.1%) and BNP (69.8%) responder groups. Hypertension was the most prevalent comorbidity, affecting over 70% of the cohort, with a statistically significant higher frequency among BNP responders (87.3%, *p* = 0.036). The prevalence of diabetes mellitus and chronic kidney disease was comparable across all groups. Ischemic heart disease was significantly more common among EF non-responders (45.8%, *p* = 0.023), while smoking was more prevalent among BNP responders (25.4%, *p* = 0.017). Biochemical parameters such as HbA1c and hematocrit showed statistically significant differences between the groups, with *p*-values of 0.046 and 0.003, respectively. Beta-blocker and diuretic use were generally balanced between EF-based groups. However, in BNP-based subgroups, beta-blockers were used by 61.9% of responders, while diuretics were more frequently prescribed to non-responders (36.8%), as shown in Table 1 and Table S1.

### 3.2. Genotypic Distribution and Ethnic Stratification

Of the 120 enrolled patients, high-quality DNA was obtained from 99 individuals. For BDNF rs6265, genotyping in 83 patients showed CC in 48 (57.83%), CT in 33 (39.76%), and TT in 2 (2.41%), with a T allele MAF of 21.08%. The BDNF CC refers to the wild-type genotype (homozygous major allele), CT to the heterozygous variant, and TT to homozygous variant (Figures S1–S3).

For ATP2A2 rs1860561, genotyping was performed in 68 patients. The GG genotype was observed in 58 patients (85.29%), and GA + AA in 10 patients (14.71%), with a minor allele frequency (MAF) of 7.35% for the A allele, as shown in Table 2. Ethnic breakdown revealed marked differences. Among them, ATP2A2 GG represents the wild-type genotype, GA is heterozygous, and AA is the homozygous variant.

**Figure 2:**
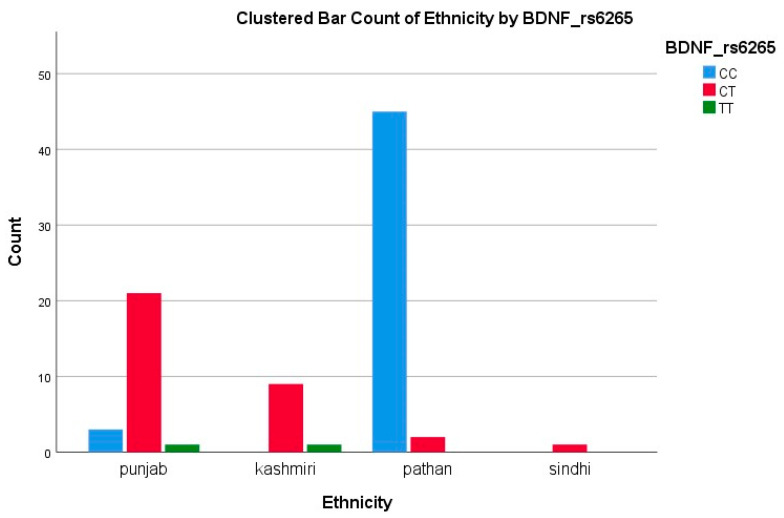
Clustered bar count of ethnicity BDNF (rs6265).

**Figure 3:**
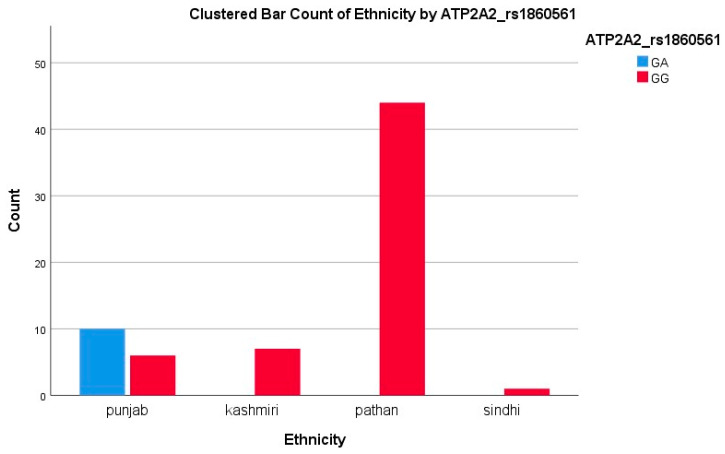
Clustered bar count of ethnicity ATP2A2 (rs1860561).

Among Pathans (*n* = 47), 45 (95.7%) had GG (ATP2A2) and CC (BDNF), indicating a low variant frequency. In Punjabis (*n* = 25), 10 (40%) had GA (ATP2A2) and 21 (84%) had CT/TT (BDNF), showing a high level of polymorphism. All Kashmiris (*n* = 10) were GG (ATP2A2), while nine (90%) had CT/TT (BDNF). The only Sindhi (*n* = 1) had GG and CT genotypes. The A allele of ATP2A2 was restricted to Punjabis, while the T allele of BDNF was more dispersed. The high T allele frequency among Punjabis/Kashmiris may reflect a greater burden of Met-carriers, suggesting a potential ethnic disparity in the SGLT2i response, consistent with our polymorphism results showing reduced response rates in these groups, as represented in Figure 2 and Figure 3.

### 3.3. Response Based on BNP Levels

#### 3.3.1. ATP2A2rs1860561

**Figure 4:**
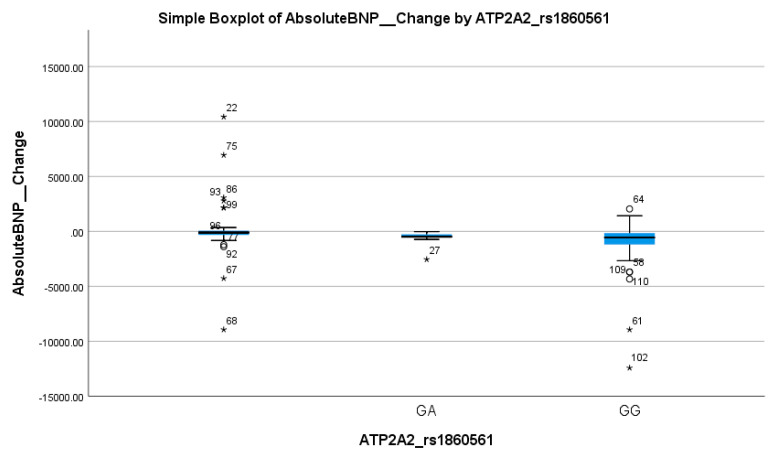
Simple boxplot of absolute change in Brain Natriuretic Peptide (Absolute BNP Change) by ATP2A2_rs1860561 genotype. The box represents the interquartile range (IQR), with the horizontal line indicating the median. Whiskers extend to the most extreme data points within 1.5 × IQR. Open circles (○) indicate mild outliers (1.5–3 × IQR), and asterisks (*) indicate extreme outliers (>3 × IQR). Only GA and GG genotypes were observed in the study population; no individuals with the AA genotype were present.

When stratified by the BNP response, no statistically significant difference was found in the distribution of ATP2A2 genotypes (*p* = 0.22), as shown in Table 2. The GG genotype was prevalent in both responders (82.4%) and non-responders (94.1%). Logistic regression analysis supported this lack of association, showing no significant effect (OR = 0.14, 95% CI: 0.01–1.7; *p* = 0.128), as shown in Table 3 and Table 4. Thus, ATP2A2 rs1860561 does not appear to influence the BNP-based treatment response. These findings suggest that the presence of the A allele has a minimal clinical impact on BNP reduction following empagliflozin therapy. This interpretation is supported by the corresponding boxplot in Figure 4, which shows overlapping interquartile ranges and median BNP changes near zero for both genotypes, without a clear directional trend.

#### 3.3.2. BDNFrs6265

**Figure 5:**
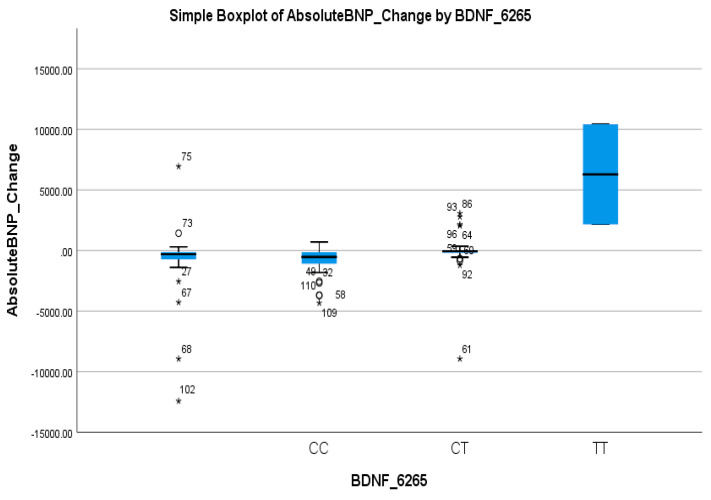
Simple boxplot of absolute change in Brain Natriuretic Peptide (Absolute BNP Change) by BDNF_6265 genotype. The box represents the interquartile range (IQR), with the horizontal line indicating the median. Whiskers extend to the most extreme data points within 1.5 × IQR. Open circles (○) indicate mild outliers (1.5–3 × IQR), and asterisks (*) indicate extreme outliers (>3 × IQR). All three genotypes (CC, CT, and TT) were observed in the study population.

In contrast, BDNF rs6265 showed a strong association with the BNP response. The CT + TT genotype was significantly more common in non-responders (66.7%) than in responders (17.1%) (*p* < 0.001), as shown in Table 2. Logistic regression confirmed the predictive value of this variant, with the CC genotype associated with a markedly higher likelihood of a BNP response (OR = 7.7, 95% CI: 2.4–24.65; *p* < 0.001), as shown in Table 4. These findings highlight the T allele as a potential marker of a poor BNP response. Table 2 shows a clear inverse relationship between the T allele frequency and BNP response, reinforcing its clinical relevance. This was visually reflected in the boxplot in Figure 5, where the TT genotype group exhibited a markedly higher median and wider spread of BNP increases compared to the CC and CT groups, further supporting the T allele’s association with reduced therapeutic efficacy.

### 3.4. Response Based on Ejection Fraction (EF)

#### 3.4.1. ATP2A2rs1860561

**Figure 6:**
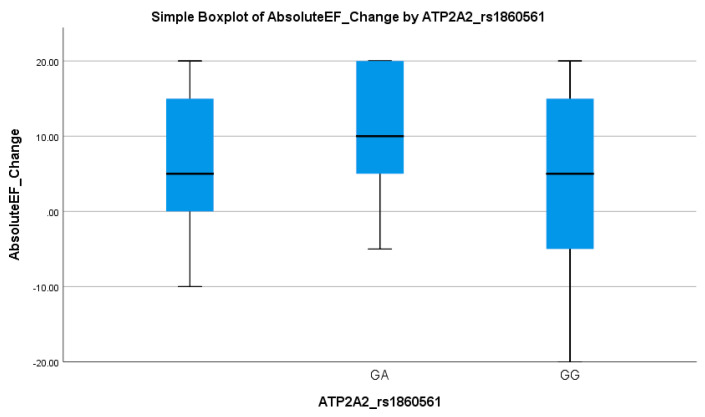
Simple boxplot of absolute change in ejection fraction (Absolute EF Change) by ATP2A2_rs1860561 genotype. The box represents the interquartile range (IQR), with the horizontal line indicating the median. Whiskers extend to the most extreme data points within 1.5 × IQR. Two genotypes (GA and GG) were observed in the study population.

A significant association was observed between ATP2A2 genotypes and the EF response (*p* = 0.040), given in Table 3. The GG genotype was more frequent in EF responders (90.5%) compared to non-responders (76.9%). Logistic regression showed a meaningful association, with the GG genotype predicting better EF improvement (OR = 5.97, 95% CI: 2.04–17.46; *p* = 0.001), as shown in Table 4. This suggests a favorable cardiac response linked to the GG variant. Table 3 demonstrates a consistent pattern where carriers of the A allele exhibit less EF improvement. This trend is visually supported by the boxplot in Figure 6, which shows that GA carriers had a wider range and slightly higher median EF change, but with overlapping interquartile ranges compared to GG homozygotes. This suggests a moderate genotype influence on the EF response, with GG showing more consistent and stable improvement.

#### 3.4.2. BDNFrs6265

**Figure 7:**
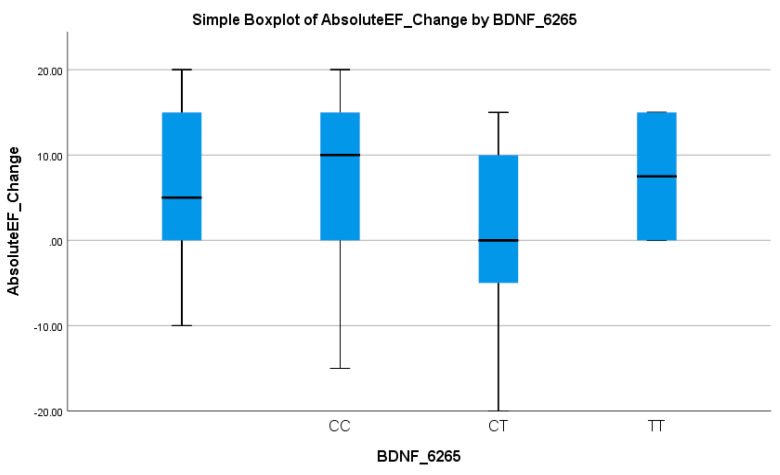
Simple boxplot of absolute change in ejection fraction (Absolute EF Change) by BDNF_6265 genotype. The box represents the interquartile range (IQR), with the horizontal line indicating the median. Whiskers extend to the most extreme data points within 1.5 × IQR. All three genotypes (CC, CT, and TT) were observed in the study population.

Similarly, the BDNF rs6265 polymorphism was associated with an EF-based response. The CT + TT genotype was present in 64.1% of non-responders versus only 22.7% of responders (*p* = 0.028), as given in Table 3. Logistic regression indicated that the CC genotype significantly increased the odds of an EF response (OR = 5.97, 95% CI: 2.04–17.46; *p* = 0.001), as shown in Table 4. These results suggest that the T allele is associated with reduced EF improvement, reinforcing its potential as a negative prognostic marker. Table 3 underscores the role of the T allele as a predictor of a suboptimal response in EF improvement. This pattern was visually evident in the boxplot in Figure 7, where CT and TT carriers showed lower median EF changes and narrower interquartile ranges compared to the CC genotype, supporting the allele’s link to diminished cardiac recovery.

## 4. Discussion

Empagliflozin has demonstrated a substantial reduction in heart-failure-related hospitalizations and mortality, with large-scale clinical trials reporting relative risk reductions of approximately 30–35% [20,21]. Despite this clear evidence, its impact on surrogate measures such as the left ventricular ejection fraction and B-type natriuretic peptides has been inconsistent [22]. In one study, adding a low dose of 10 mg empagliflozin to standard therapy yielded a 5–7% absolute increase in EF and significant NT-proBNP declines, yet another trial found no meaningful change in the EF or BNP levels despite similar reductions in clinical events [23]. Such discordance suggests that glycosuria and diuresis alone cannot account for inter-individual variability in cardiac remodeling or the biomarker response.

In the EF-based analysis, responders and non-responders did not differ in mean age (58.1 vs. 59.7 years; *p* = 0.362) or gender distribution (68.1% vs. 58.8% male; *p* = 0.624), echoing pooled data showing minimal age or sex effects on SGLT2 inhibitor-mediated remodeling [24]. Ischemic heart disease was significantly more prevalent among EF non-responders than responders (45.8% vs. 20.3%; *p* = 0.023), consistent with prior work demonstrating that the scar burden limits reverse remodeling [25,26].

In contrast, in the BNP-based analysis, age again showed no effect (58.5 vs. 59.2 years; *p* = 0.674), but the male sex was over-represented among responders (69.8% vs. 43.9%; *p* = 0.004), in line with subgroup findings that natriuretic peptide reductions under SGLT2 inhibition are modulated by sex [14,27,28,29]. Hypertension was more common in BNP responders (87.3% vs. 71.9%; *p* = 0.036), supporting evidence that an afterload reduction augments BNP decline and smoking history likewise in differentiated groups (25.4% vs. 8.8%; *p* = 0.017), reflecting tobacco’s hemodynamic effects [30,31]. Although HbA1c differed modestly between responders and non-responders (7.73 ± 2.19% vs. 7.11 ± 1.86%; *p* = 0.046), no single clinical variable or their combination accounted for the high non-response rate, pointing to genetic factors as key modulators of individual benefits.

An inherited genetic variation emerged as a powerful predictor of therapeutic benefit. The Val66Met polymorphism in the BDNF rs6265 proved the strongest single predictor carriers of at least one Met allele (CT or TT genotype) which accounted for 66.7% of BNP non-responders versus 17.1% of responders (*p* < 0.001), and 64.1% of EF non-responders versus 22.7% of responders (*p* = 0.028). These effect sizes surpass those of any clinical covariate. Mechanistic studies have shown that the Met variant disrupts activity-dependent BDNF secretion and intracellular trafficking, impairing pro-survival and pro-angiogenic signaling in cardiomyocytes, leading to worse remodeling and function in animal models [14,32]. Clinically, reduced circulating BDNF levels are associated with more advanced stages of heart failure and a worse prognosis [33]. Our findings extend these observations to SGLT2 inhibition, suggesting that intact BDNF signaling is necessary for maximal reverse remodeling under empagliflozin.

Among 83 BDNF-genotyped patients (25 Punjabi, 10 Kashmiri, 47 Pathan, and 1 Sindhi), the CT genotype was found in 21/25 Punjabis (84%), 9/10 Kashmiris (90%), 2/47 Pathans (4%), and 0/1 Sindhi (0%). Conversely, the CC genotype predominated in Pathans (45/47, 96%), but was present in only 3/25 Punjabis (12%) and 0/10 Kashmiris. This stark inter-ethnic variability mirrors the 20–65% range reported for CYP2C19 loss-of-function alleles in antiplatelet studies and highlights the need for population-specific pharmacogenetic strategies [34].

A second locus, the intronic ATP2A2 rs1860561 variant, was significantly associated with EF, but not the BNP response (*p* = 0.040 vs. *p* = 0.22). GG homozygotes comprised 90.5% of EF responders versus 76.9% of non-responders; GA carriers were over-represented among non-responders. SERCA2a, encoded by ATP2A2, mediates calcium reuptake in cardiomyocytes, and its dysfunction is a hallmark of systolic heart failure [35,36]. Recent preclinical work suggests that empagliflozin may enhance SERCA2a expression and activity indirectly by improving myocardial energetics [37,38]. Our data imply that A-allele carriers derive less contractile and remodeling benefit. Once again, the A allele was confined to Punjabi patients (58.8% GA), underscoring an ethnicity-specific pharmacogenetic effect that may guide adjunctive therapy aimed at calcium handling.

Regression analysis also confirmed the dominant influence of genetic polymorphisms over clinical factors. For BDNF rs6265, individuals with the CC genotype had significantly higher odds of an EF (OR: 5.97; *p* = 0.001) and BNP response (OR: 7.7; *p* < 0.001) compared to CT/TT carriers. ATP2A2 rs1860561 was associated with an EF response (OR: 0.09; *p* = 0.048), though not with BNP (*p* = 0.128). These associations remained significant after adjusting for ischemic heart disease, hypertension, smoking, and gender, highlighting the stronger predictive value of the genotype over traditional clinical variables. These results have particular relevance in South Asian populations, where marked inter-ethnic differences in allele frequencies were observed. The high prevalence of the BDNF rs6265 T allele and ATP2A2 rs1860561 A allele in certain ethnic subgroups (e.g., Punjabis, Kashmiris) underscores the need for South Asia-specific pharmacogenetic profiling. Incorporating genotyping into routine clinical care could improve precision therapy for heart failure in this under-represented population [39].

Together, these findings argue for the integration of pharmacogenetic screening into heart failure management. Genotyping BDNF rs6265 and ATP2A2 rs1860561 before initiating empagliflozin could stratify patients by their likelihood of benefit and inform personalized treatment strategies, whether that involves combining SGLT2 inhibition with neurotrophic enhancers in Met carriers or calcium-handling support in ATP2A2 A-allele individuals.

## 5. Conclusions

In conclusion, our data demonstrate that genetic polymorphisms in BDNF and ATP2A2, far more than traditional clinical or demographic factors, explain a substantial portion of the variability in the EF and BNP response to empagliflozin. Pronounced ethnic disparities in allele frequencies highlight the urgent need for tailored pharmacogenetic strategies in diverse HF populations. Future prospective, multicenter studies incorporating functional validation and extended clinical follow up will be essential to translate these insights into precision-guided heart failure therapy. These results support genotype-guided treatment strategies and highlight the need for pharmacogenetic infrastructure in South Asia.

## Figures and Tables

**Table 1:** Characteristics of the study population.

Total No. of Patients (*n* = 120)	Ejection Fraction		*p*-Value	Brain Natriuretic Peptide		*p*-Value
	Responders (*n* = 69)	Non- Responders (*n* = 51)		Responders (*n* = 63)	Non- Responders (*n* = 57)	
Demographic characteristics mean ± SD						
Age (Years)	58.14± 9.04	59.66± 9.06	0.362	58.46 ± 9.05	59.16 ± 9.02	0.674
Gender Male Female	47 (68.1%) 22 (31.9%)	30 (58.8%) 26 (41.7%)	0.624	44 (69.8%) 19 (30.2%)	25 (43.9%) 32 (56.1%)	0.004
Medical history/Comorbidities *n* (%)						
Hypertension Yes No	54 (78.3%) 42 (21.7%)	42 (82.4%) 9 (17.6%)	0.580	55 (87.3%) 8 (12.7%)	41 (71.9%) 16 (28.1%)	0.036
Diabetes Mellitus Yes No	42 (60.9%) 27 (39.1%)	31 (60.8%) 20 (39.2%)	0.992	39 (61.9%) 24 (38.1%)	34 (59.6%) 23 (40.4%)	0.800
Chronic Kidney Disease (CKD) Yes No	22 (31.9%) 47 (68.1%)	13 (25.5%) 38 (74.5%)	0.446	14 (22.2%) 49 (77.8%)	21 (36.8%) 36 (63.2%)	0.078
Ischemic Heart Disease (IHD) Yes No	14 (20.3%) 20 (79.7%)	55 (45.8%) 31 (25.8%)	0.023	17 (27.0%) 46 (73.0%)	17 (29.8%) 40 (70.2%)	0.730
Smoking Yes No	12 (10.0%) 57 (82.6%)	20 (39.2%) 42 (82.4%)	0.971	16 (25.4%) 47 (74.6%)	5 (8.8%) 52 (91.2)	0.017
Medications at baseline *n* (%)						
Beta-Blockers Yes No	42 (60.9%) 27 (39.1%)	31 (60.8%) 20 (39.1%)	0.992	39 (61.9%) 24 (38.1%)	34 (42.1%) 23 (57.9%)	0.800
Diuretics Yes No	22 (31.9%) 47 (68.1%)	13 (25.5%) 38 (74.5%)	0.446	14 (22.2%) 49 (77.8%)	21 (36.8%) 36 (63.2%)	0.078

EF, Ejection Fraction; BNP, Brain Natriuretic Peptide; DM, Diabetes Mellitus; CKD, Chronic Kidney Disease; IHD, Ischemic Heart Disease; HTN, Hypertension; *p*-value, Significance level.

**Table 2:** Baseline laboratory characteristics of the investigated population with respect to response in BNP.

Variable		Patients *n* = 120	Response with Respect to BNP		*p*-Value
			Responders (*n* = 63)	Non- Responders (*n =* 57)	
Laboratory Findings	Triglycerides (mg/dL)	162.30 ± 87.77	171.22 ± 103.69	153.8 ± 64,65	0.264
	HDL (mg/dL)	38.03 ± 7.96	38.70 ± 8.34	37.30 ± 7.52	0.338
	LDL (mg/dL)	108.61 ± 36.59	106.68 ± 36.17	110.74 ± 37.26	0.547
	Total Cholesterol (mg/dL)	173.03 ± 41.53	173.97 ± 40.62	172.0 ± 48.4	0.797
	HbA1c	7.44 ± 2.07	7.73 ± 2.193	7.11 ± 1.86	0.099
	Hematocrit	37.01 ± 11.44	34 ± 6.25	40 ± 14.62	0.003
	Creatinine (mg/dL)	1.51 ± 0.76	1.48 ± 0.71	1.53 ± 0.82	0.772
	eGFR (mL/min/1.73 m^2^)	61.55 ± 22.11	61.773 ± 21.1	61.39 ± 23.1	0.926
Heart failure characteristics mean ± SD	EF at baseline	34.00 ± 8.03	33.81 ± 7.92	34.21 ± 8.23	0.786
BNP Biomarker					
	BNP at baseline (pg/mL)	1458.02 ± 1886.70	1768.4 ± 2280.95	1114.0 ± 1255.35	0.058
ATP2A2 rs1860561 *n* = 68	GG	58 (85.29%)	42 (72.4%)	16 (94.12%)	0.22
	GA + AA	10 (14.71%)	9 (15.2%)	1 (5.88%)	
	G Allele	126 (92.65%)	101 (91.8%)	25 (96.2%)	
	A Allele	10 (7.35%)	9 (8.2%)	1 (3.8)	
BDNF rs6265 *n* = 83	CC	48 (57.83%)	34 (82.9%)	14 (33.3%)	<0.001 **
	CT + TT	35 (42.17%)	7 (17.1%)	28 (66.7%)	
	C Allele	131 (78.92%	75 (91.5%)	56 (66.7%)	
	T Allele	35 (21.08%)	7 (8.5%)	28 (33.3%)	

BNP, Brain Natriuretic Peptide; HDL, High-Density Lipoprotein; LDL, Low-Density Lipoprotein; HbA1c, Glycosylated Hemoglobin A1C; eGFR, Estimated Glomerular Filtration Rate; EF, Ejection Fraction; IHD, Ischemic Heart Disease; *p*-value, Significance level; ** statistically significant. *p*-values indicate genotype distribution differences between responders and non-responders within each gene subgroup (ATP2A2 and BDNF).

**Table 3:** Baseline laboratory characteristics of the investigated population with respect to response in ejection fraction.

Variable		Patients *n* = 120	Response with Respect to Ejection Fraction		*p*-Value
			Responders (*n=* 69)	Non-Responders (*n=* 51)	
Laboratory Findings	Triglycerides (mg/dL)	162.30 ± 87.77	158.6 ± 99.82	160.88 ± 69.20	0.655
	HDL (mg/dL)	38.03 ± 7.96	37.5 ± 7.86	38.75 ± 8.10	0.402
	LDL (mg/dL)	108.61 ± 36.59	107.4 ± 36.11	110.7 ± 37.50	0.630
	Total Cholesterol (mg/dL)	173.03 ± 41.53	173.4 ± 39.31	172.4 ± 44.73	0.892
	HbA1c	7.44 ± 2.07	7.1 ± 1.85	7.8 ± 2.85	0.046
	Hematocrit	37.01 ± 11.44	36.3 ± 9.096	37.9 ± 14.05	0.448
	Creatinine (mg/dL)	1.51 ± 0.76	1.49 ± 0.73	1.52 ± 0.78	0.799
	eGFR (mL/min/1.73 m^2^)	61.55 ± 22.11	61.2 ± 20.29	61.9 ± 24.37	0.864
Heart failure characteristics mean ± SD					
	EF at baseline	34.00 ± 8.03	33.4 ± 7.08	34.7 ± 9.18	0.410
BNP Biomarker					
	BNP at baseline (pg/mL)	1458.02 ± 1886.70	1334 ± 1658.5	1625 ± 2163.5	0.405
ATP2A2 rs1860561 *n* = 68	GG	58 (85.29%)	29 (90.5%)	29 (76.9%)	0.040
	GA + AA	10 (14.71%)	9 (9.5%)	1 (23.1%)	
	G Allele	126 (92.65%)	67 (53.2%)	59 (46.8%)	
	A Allele	10 (7.35%)	9 (46.8)	1 (44.4)	
BDNF rs6265 *n* = 83	CC	48 (57.83%)	34 (77.3%)	14 (35.9%)	0.028 **
	CT + TT	35 (42.17%)	10 (22.7%)	25 (64.1%)	
	C Allele	131 (78.92%)	78 (88.6%)	53 (67.9%)	
	T Allele	35 (21.08%)	10 (11.4%)	25 (32.1%)	

BNP, Brain Natriuretic Peptide; HDL, High-Density Lipoprotein; LDL, Low-Density Lipoprotein; HbA1c, Glycosylated Hemoglobin A1C; eGFR, Estimated Glomerular Filtration Rate; EF, Ejection Fraction; IHD, Ischemic Heart Disease; *p*-value, Significance level; **, statistically significant *p*-value,. *p*-values indicate genotype distribution differences between responders and non-responders within each gene subgroup (ATP2A2 and BDNF).

**Table 4:** Binary logistic regression analysis of ATP2A2 rs1860561 and BDNF rs6265 genotype distribution with respect to BNP and ejection fraction.

Genotype	EF Responders *n* (%)	EF Non- Responders *n* (%)	B	OR (95% CI)	*p*-Value	BNP Responders *n* (%)	BNP Non- Responders *n* (%)	B	OR (95% CI)	*p*- Value
ATP2A2 rs1860561 (*n* = 68)										
GG (*n* = 58)	29 (76.3%)	29 (96.7%)	3.91	0.09 (0.01–0.97)	0.048	42 (82.4%)	16 (94.1%)	2.31	0.14 (0.01–1.7)	0.128
GA + AA (*n* = 10)	9 (23.7%)	1 (3.3%)				9 (17.6%)	1 (5.9%)			
BDNF rs6265 (*n* = 83)										
CC (*n* = 48)	34 (77.3%)	14 (35.9%)	10.64	5.97 (2.04–17.46)	0.001	34 (82.9%)	14 (33.3%)	12.19	7.7 (2.4–24.65)	<0.001
CT + TT (*n* = 35)	10 (22.7%)	25 (64.1%)				7 (17.1%)	28 (66.7%)			

B, regression coefficient; OR, odds ratio; CI, Confidence interval; *p*-value, significance level.

## Data Availability

All data generated or analyzed during this study are included in this article. Further inquiries can be directed to the corresponding author.

## Supplementary Materials

The following supporting information can be downloaded at: https://www.mdpi.com/article/10.3390/biomedicines13092095/s1, Figure S1. The steps involved in DNA extraction protocol; Figure S2. Gel electrophoresis image displaying the separation of DNA fragments based on size. Figure S3. rs6265 polymorphism (*BDNF* Val66Met-Variant) Sequencing Chromatogram of Patients with C/T Polymorphism at position c 460. Figure S4. rs1860561 polymorphism *ATP2A2* Sequencing Chromatogram of Patients with G/A Polymorphism at position c162. Table S1. Characteristics of the Study Population.
