# Supplementary material for: Personalized Response to Empagliflozin in Heart Failure: Association of BDNF and ATP2A2 Variants in a South Asian Cohort
Source: Biomedicines. 2025 Aug 28;13(9):2095. doi: 10.3390/biomedicines13092095 (PMC12467674; doi:10.3390/biomedicines13092095)
Supplement: Supplementary file 1 [file biomedicines-13-02095-s001.zip › biomedicines-3730239-supplementary.pdf]

biomedicines

MDPI

Supplementary Material

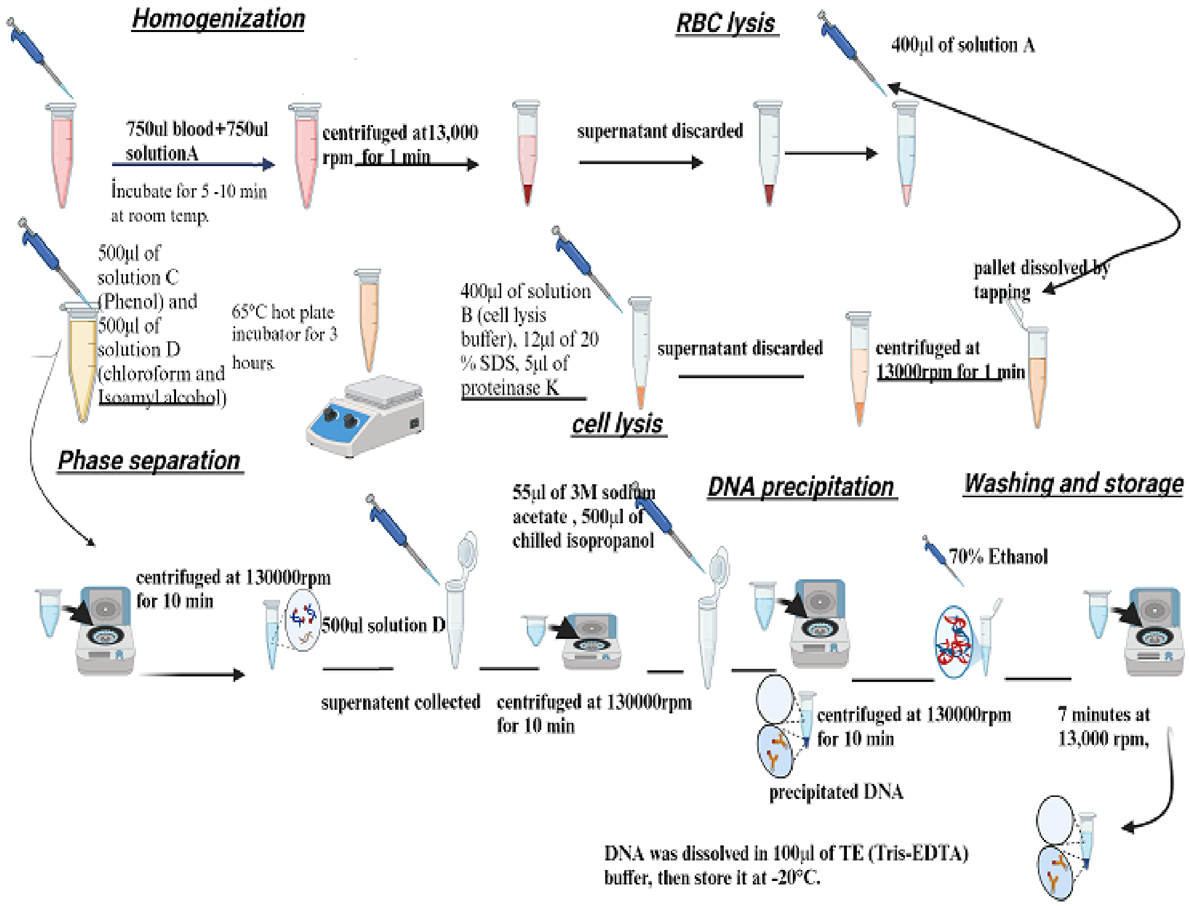

Figure S1. The steps involved in DNA extraction protocol.

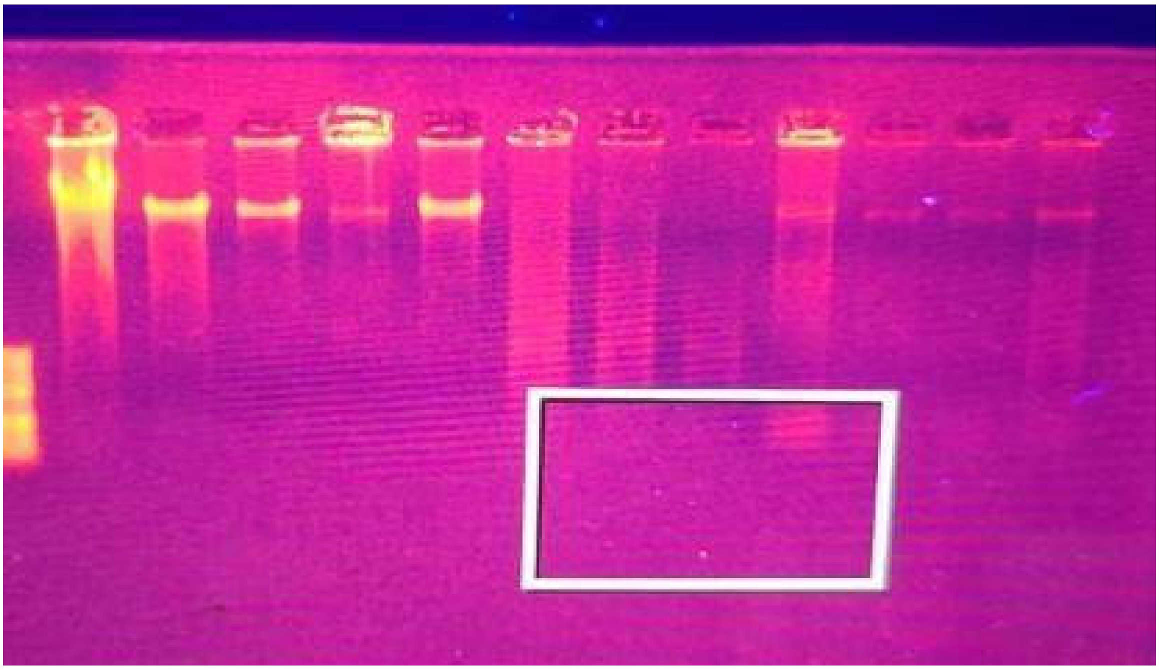

Figure S2. Gel electrophoresis image displaying the separation of DNA fragments based on size.

a

b

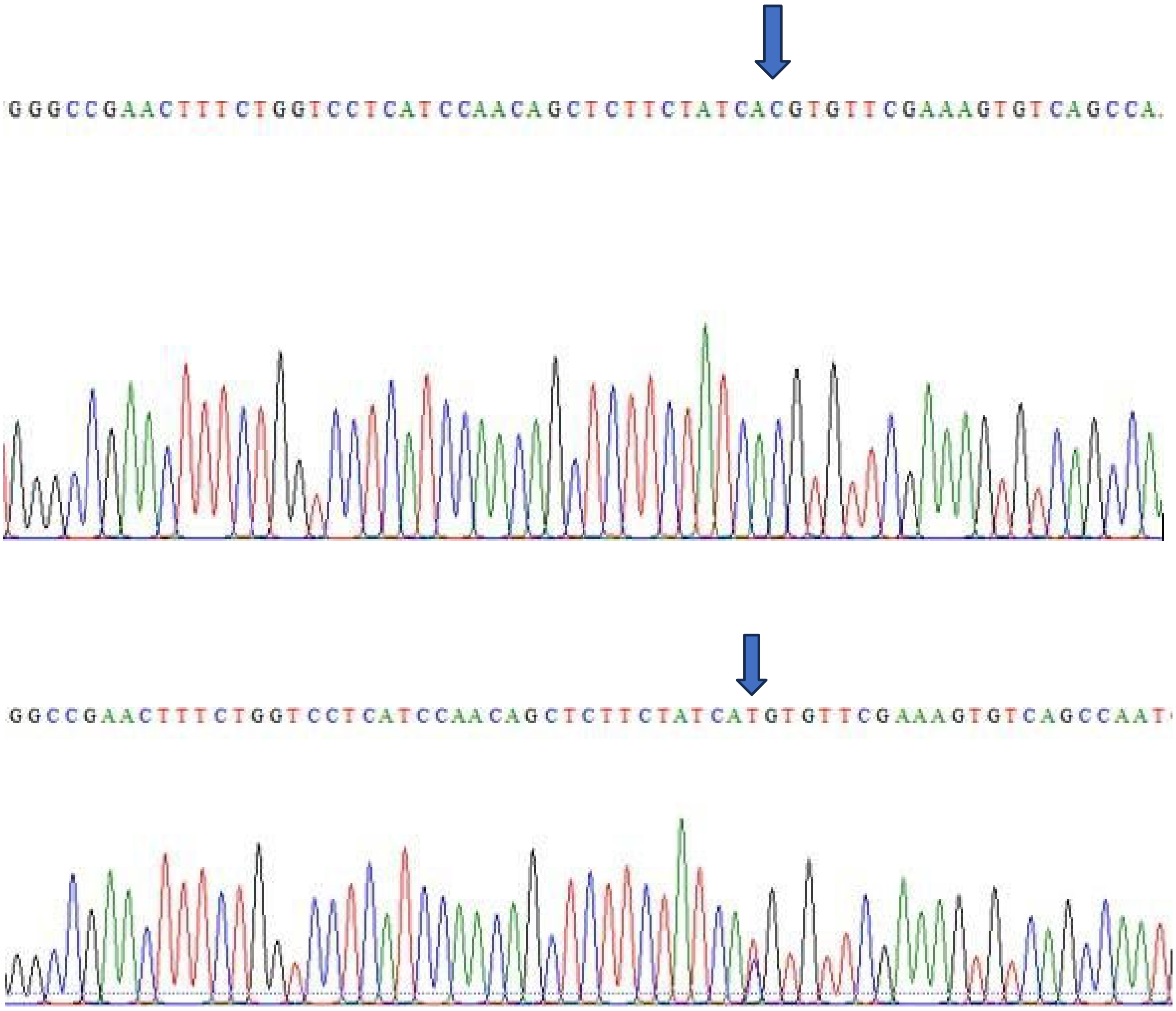

Figure S3. rs6265 polymorphism (*BDNF* Val66Met-Variant) Sequencing Chromatogram of Patients with C/T Polymorphism at position c 460 a) Homozygous Type b) Heterozygous type.

a

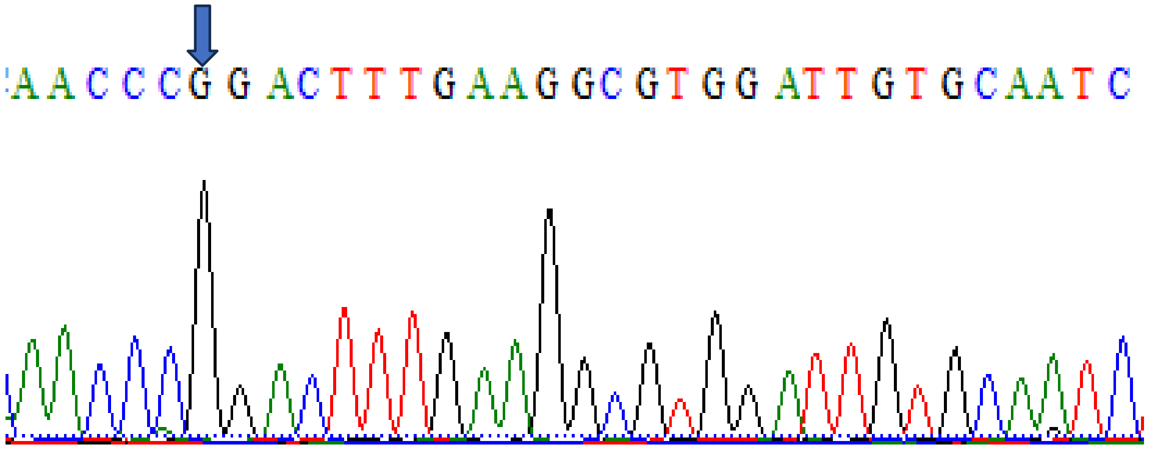

b

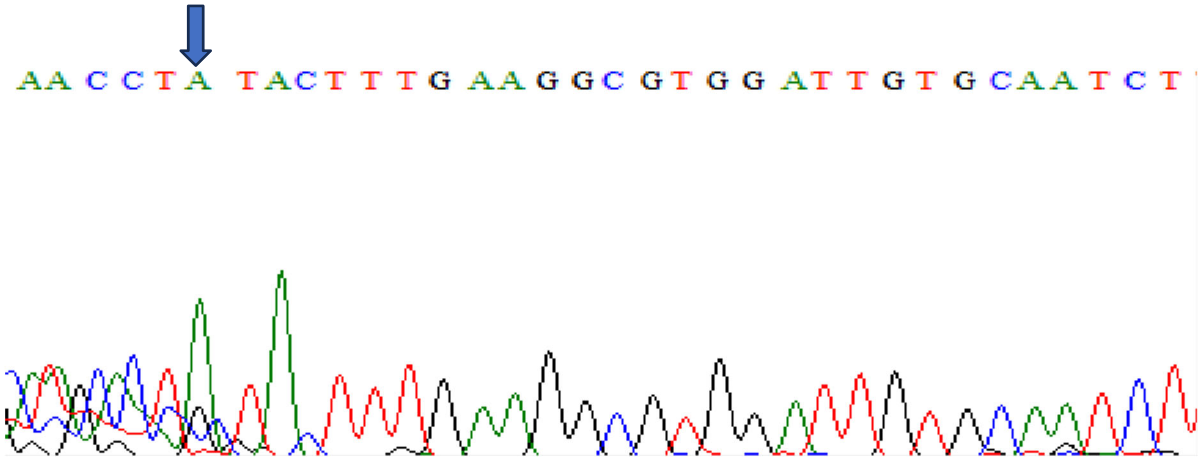

Figure S4. rs1860561 polymorphism *ATP2A2* Sequencing Chromatogram of Patients with G/A Polymorphism at position c162 a) Homozygous Type b) Heterozygous type.

Table S1. Characteristics of the Study Population.

Characteristic	Value or mean ± SD	% or Range
**Demographic characteristics**		
Age (Years)	58.79 ± 9.01	36 - 79
Gender		
Male	69	54.8
Female	51	40.5
**Medical history / Comorbidities**		
Hypertension		
Yes	96	76.2
No	24	19
Diabetes Mellitus		
Yes	73	57.9
No	47	37.3
Chronic Kidney Disease (CKD)		
Yes	35	27.8
No	85	67.5
Ischemic Heart Disease (IHD)		
Yes	34	27
No	86	68.3
Smoking		
Yes	21	16.7
No	99	78.6
**Laboratory values**		
Triglycerides (mg/dL)	162.30 ± 87.77	20 - 850
HDL (mg/dL)	38.03 ± 7.96	24 – 58
LDL (mg/dL)	108.61 ± 36.59	34 - 201
Total Cholesterol (mg/dL)	173.03 ± 41.53	97 - 254
HbA1c	7.44 ± 2.07	5.10 - 15
Hematocrit	37.01 ± 11.44	24 – 122.63
Creatinine (mg/dL)	1.51 ± 0.76	0.50 – 4.60
GFR (mL/min/1.73 m^2)	61.55 ± 22.11	0.55–117.11
**Heart failure characteristics**		
EF at baseline	34.00 ± 8.03	20 - 60
**BNP Biomarker**		
BNP at baseline (pg/mL)	1458.02 ± 1886.70	65.40 - 12856
**Medications at baseline**		
Beta-Blockers		
Yes	73	57.9
No	47	37.3
Diuretics		
Yes	35	27.8
No	85	67.5
